# Vaccination coverage for seasonal influenza among residents and health care workers in Norwegian nursing homes during the 2012/13 season, a cross-sectional study

**DOI:** 10.1186/1471-2458-14-434

**Published:** 2014-05-09

**Authors:** Horst Bentele, Marianne R Bergsaker, Siri Helene Hauge, Jørgen V Bjørnholt

**Affiliations:** 1 Department of Infection Control Epidemiology, Norwegian Institute of Public Health, Oslo, Norway; 2 Department of Vaccines, Norwegian Institute of Public Health, Oslo, Norway; 3 European Programme for Intervention Epidemiology Training (EPIET), European Centre for Disease Prevention and Control (ECDC), Stockholm, Sweden

## Abstract

**Background:**

WHO has set a goal of 75% vaccination coverage (VC) for seasonal influenza for residents and also recommends immunization for all healthcare workers (HCWs) in nursing homes (NHs). We conducted a cross-sectional study to estimate the VC for seasonal influenza vaccination in Norwegian NHs in 2012/2013 since the VC in NHs and HCWs is unknown.

**Methods:**

We gathered information from NHs concerning VC for residents and HCWs, and vaccination costs for HCWs, using a web-based questionnaire. We calculated VC among NH residents by dividing the number of residents vaccinated by the total number of residents for each NH. VC among HCWs was similarly calculated by dividing the number of HCWs vaccinated by the total number of HCWs for each NH. The association between VC and possible demographic variables were explored.

**Results:**

Of 910 NHs, 354 (38.9%) responded. Median VC per NH was 71.7% (range 0-100) among residents and 0% (range 0-100) among HCWs, with 214 (60%) NHs reporting that none of their HCWs was vaccinated. Median VC for HCWs in NHs with an annual vaccination campaign was 0% (range 0-53), compared to when they did not have an annual vaccination campaign 0% (range 0-12); the distributions in the two groups differed significantly (Mann–Whitney U, P = 0.006 two tailed).

**Conclusion:**

Median influenza VC in Norwegian NHs was marginally lower than recommended among residents and exceptionally low among HCWs. The VC in HCWs was significantly higher when NHs had an annual vaccination campaign. We recommend that NHs implement measures to increase VC among residents and HCWs, including vaccination campaigns and studies to identify potential barriers to vaccination.

## Background

Residents in nursing homes (NHs) and long-term care facilities (LTCF) are at higher risk of severe respiratory tract infections owing to old age, the number of underlying illnesses, and close living conditions [1-3]. The main vaccine- preventable respiratory tract infection is caused by the influenza virus. The infection can be severe and even lethal, and viral infections often predispose sufferers to bacterial secondary infections and complications [4]. Further, although the lethality of influenza infection is low, the resulting reduced general condition following infection is of major importance in the elderly. Accordingly, the World Health Organization (WHO) defines NHs residents as a risk group for severe influenza and recommends vaccination coverage (VC) of at least 75%. The main goals are to reduce risk of severe disease and to prevent outbreaks. The WHO recommendation also includes influenza vaccination of health care workers (HCWs) in order to prevent introduction of the disease into healthcare institutions [5,6]. The Norwegian guidelines for infectious disease control follow the WHO recommendations relating to seasonal influenza, the aim being to achieve a minimum VC of 75% in Norwegian NHs [7]. Seasonal influenza vaccination for residents in NHs is reimbursed by the state in Norway. This is in contrast to influenza vaccination of HCWs, for whom each employer decides whether it is to be given free of charge. The yearly influenza vaccination is normally conducted during October and November [7].

The Vaccine European New Integrated Collaboration Effort (VENICE) report, covering the 2011/2012 season, showed seasonal influenza VC for the general Norwegian population above 65 years and for all HCWs to be 36% and 12% respectively [8]. However, the VC among residents and HCWs in NHs is unknown. To guide development of vaccination programs for residents and HCWs in NHs, data on VC is crucial. We therefore conducted a cross-sectional study approaching all NHs in Norway in order to estimate VC for seasonal influenza vaccine among both residents and HCWs. In addition, we investigated whether there was an association between VC and response rate by county, yearly vaccination campaigns among residents and HCWs, free-of-charge vaccination of HCWs, NH size (number of residents) and geographic location.

## Methods

### Population and data collection

In December 2012 we invited NH managers and NH physicians to participate in an electronic survey. The invitation was sent to all 429 municipalities in Norway with the instruction to forward the e-mail to all the NHs located in the respective municipality. After six weeks a reminder was sent to the same e-mail-addresses. The total number of residents in NHs in the different counties was obtained from Statistics Norway [9]. In 2011, 910 NHs with a total of 34,795 long-term-care residents were registered in Norway.

The questionnaire (Additional file 1) was developed in QuestBack to collect aggregated data from each NH. The questionnaire contained 10 closed questions regarding seasonal influenza vaccination of residents and HCWs. We also collected general information from the NHs, including geographic location, number of long-term-care residents, number of personnel working in direct contact with the residents, whether influenza vaccination of HCWs was given free of charge and, if not, how much personnel had to pay, and if the NHs had annual vaccination campaigns.

### Definitions and data analyses

In Norway, a NH is defined as residential facilities for elderly people with the primary purpose of providing a continuous 24-hour professional health-care service. In this study we only included long-term-care residents with registered home address at the NHs, since their medical service, including vaccination, is provided by the NH. We defined a resident as a person with registered home address at the NH. We defined an HCW as an employee at an NH who has regular physical contact with the residents. This includes doctors, nurses, auxiliary nurses, occupational therapists, physiotherapists, and students.

VC among NH residents was calculated by dividing the number of residents vaccinated by the total number of residents for each NH. Similarly, VC among HCWs was calculated by dividing the number of HCWs vaccinated by the total number of HCWs for each NH, thereby defining NH as unit of analysis. VC is presented as median per county to avoid revealing the NH identity on a municipality level. We compared the VC between NHs where HCWs had to pay for their vaccination and those who got it for free using a Mann-Whitney U-test. Similarly, we compared the VC in both residents and HCWs in NHs who had an annual vaccination campaign and those who did not. Further associations were examined by correlation analyses (Spearman rank test). The statistical analyses were done in Excel 2010 and Stata12. A p-value ≤ 0.05 was considered significant [10].

All data collected were aggregated. Complete anonymity for persons and NHs was given and therefore ethical approval in Norway not mandated. Participation in the study was voluntary.

## Results

How many of the 910 NHs actually received the invitation is unknown since distribution was by means of generic municipality e-mail addresses. However, 391 of the intended 910 invitees in Norway (43.0%) responded to our survey. We excluded 37 NHs on the basis of incomplete data; thus 354 (38.9%) NHs were included in the analysis. These 354 NHs were located in 244 (57%) of the 429 municipalities in Norway, with representatives from all 19 counties. The response rate by county ranged from 26.7% in Buskerud to 65.5% in Finnmark (Table 1).

The 354 NHs included in the study provided aggregated information on a total of 14,208 residents and 28,237 HCWs. The median number of long-term-care residents per NH was 33 (range 1-168). The median number of HCWs per NH was 60 (range 15-400). The high number of HCWs is explained by the fact that we included aggregated data about all NH HCWs in our survey, independent of NH department, since they usually rotate and hence work both in long- and short-term-care.

### Seasonal influenza vaccination coverage

**Figure 1:**
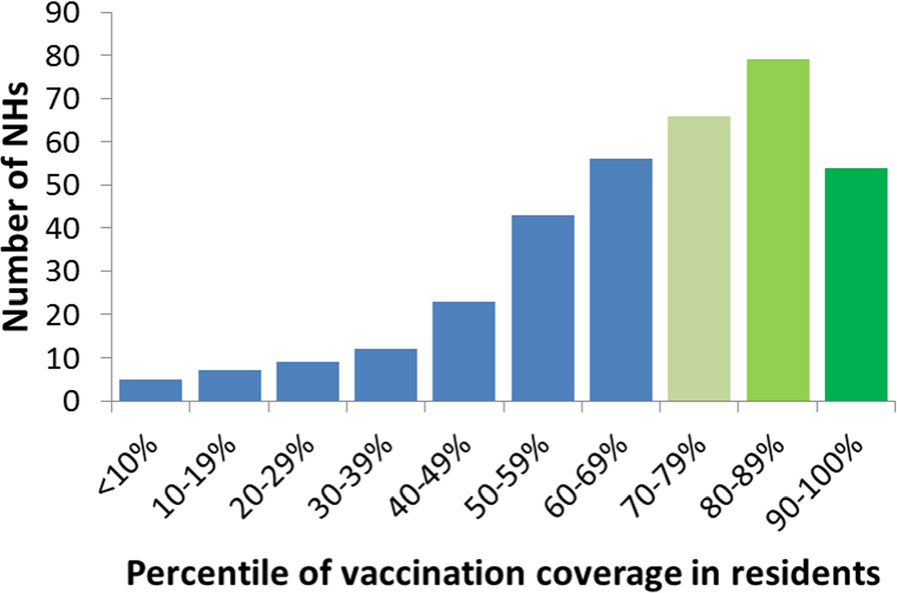
Distribution of NHs in percentile vaccination coverage.

**Figure 2:**
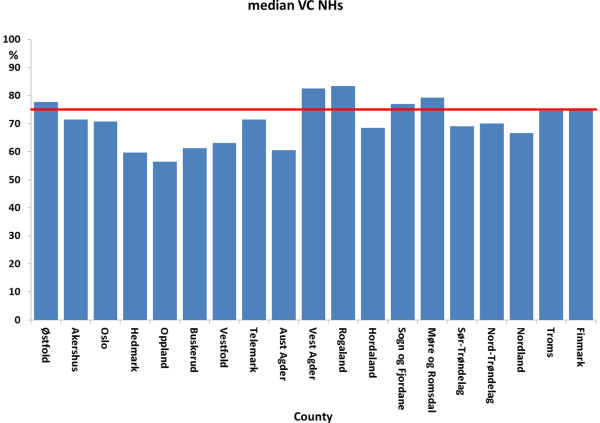
Median vaccination coverage among residents per NH in % for each county.

In the season 2012/13, 54% of the NHs had a VC among residents below 75% and in 15% of NHs VC among residents was below 50% (Figure 1), the median VC being 71.7% (range 0-100) for all participating NHs. In six counties, the median NH VC was above 75%, including one county with a VC of 83.3% (range 0-100) (Figure 2). We did not find an association between the response rate by county and vaccination coverage among residents (Spearman rank test; rho 0.35; P = 0.15).

For HCWs, the median vaccination coverage was 0% (range 0-100) (Table 1), with 214 (60%) NHs reporting that none of their HCWs was vaccinated.

**Table 1:** Response rate and vaccination coverage (VC) among long-term-care residents and HCWs of the responding NHs (n=354), by county

**County**	**Response rate (%)**	**Long term care residents**		**HCWs**	
		**Median number (range)**	**Median VC (range)**	**Median number (range)**	**Median VC (range)**
Akershus	21/66 (31.8)	32 (22–130)	71.4 (19.2-88.9)	65 (30–395)	0 (0 – 14.7)
Aust Agder	14/30 (46.7)	33 (8–66)	60.6 (0–100)	58 (33–150)	0 (0 – 6.3)
Buskerud	12/45 (26.7)	40 (20–108)	61.3 (15.4-91.9)	54 (17–400)	0 (0 – 9.6)
Finnmark	19/29 (65.5)	16 (6–40)	75.0 (18.8-100)	35 (17–85)	0 (0 – 29.4)
Hedmark	16/39 (41.0)	42 (23–110)	59.7 (22.7-100)	74 (35–247)	0 (0 – 3.0)
Hordaland	34/86 (39.5)	33 (9–143)	69.5 (25.0-100)	55 (15–300)	0.3 (0 – 22.2)
Møre og Romsdal	27/67 (40.3)	28 (5–76)	79.3 (28.1-100)	49 (12–110)	0 (0 – 18.2)
Nordland	22/74 (29.7)	29 (9–83)	66.7 (10.7-100)	60 (15–140)	2.5 (0 – 42.9)
Nord-Trøndelag	15/36 (41.7)	32 (8–86)	70.0 (18.5-92.1)	50 (22–200)	0 (0 – 5.7)
Oppland	11/39 (28.2)	40 (25–81)	54.8 (0–100)	90 (55–154)	0 (0 – 3.0)
Oslo	20/58 (34.5)	73 (11–168)	70.8 (37.5-100)	110 (16–400)	1.7 (0 – 8.6)
Rogaland	38/72 (52.8)	31 (1–120)	83.3 (0–100)	70 (15–300)	0 (0 – 53.3)
Sogn og Fjordane	16/34 (47.1)	33 (16–68)	77.0 (29.4-98.1)	60 (24–110)	4.4 (0 – 25.0)
Sør-Trøndelag	25/56 (44.6)	34 (13–91)	69.1 (27.7-100)	50 (25–158)	0 (0 – 33.3)
Telemark	11/30 (36.7)	38 (14–74)	71.4 (44.4-93.3)	60 (35–140)	0 (0 – 28.6)
Troms	12/43 (27.9)	28 (8–76)	74.5 (35.2-100)	59 (19–220)	0 (0 – 22.2)
Vest Agder	10/33 (30.3)	22 (6–106)	82.6 (58.3-100)	35 (20–170)	0 (0 – 13.8)
Vestfold	13/34 (38.2)	42 (12–80)	63.2 (40.0-93.3)	68 (39–195)	0 (0 – 6.1)
Østfold	19/39 (48.7)	51 (14–121)	77.7 (5.4-100)	110 (25–170)	0 (0 – 16.7)

* The true denominator is not known; the figures underestimate the true coverage, see Discussion.

### Financing influenza vaccination for HCWs

Of the 354 responding NHs, 165 (46.6%) provided information on vaccination cost for HCWs. The vaccination was free of charge in 143 (86.7%) NHs [median vaccination coverage for employees free of charge 3.6% (min 0, max 53.3%)] while in 22 NHs employees had to pay for their own influenza vaccination [median vaccination coverage for employees who had to pay 3.6% (min 0, max 13.3%; P = 0.47)]. The price varied between 1.50-25 Euros.

### Vaccination promotion

Of the 354 participating NHs, 316 (89.3%) answered that they had performed an annual vaccination campaign, 38 did not promote annual vaccination campaigns or the issue was unknown to them. Median VC for HCWs in the NHs having an annual vaccination campaign was 0% (range 0-53), compared to when they did not have an annual vaccination campaign 0% (range 0-12); the distributions in the two groups differed significantly (Mann–Whitney U, P = 0.006 two tailed). Our data showed no difference between the VC of residents in NHs with an annual vaccination campaign (median 72.4) and those without a vaccination campaign (median 68.5; P = 0.27).

## Discussion

To the best of our knowledge, this is the first study to aim at providing data on VC for seasonal influenza vaccine among residents and HCWs in Norwegian NHs.

In around half of the NHs, VC for seasonal influenza vaccination among residents was below the 75% recommended by WHO and laid down in National guidelines. However, the VC of NH residents was markedly higher than that in the general population above 65 years of age [8]. This may reflect both the impact of the vaccination recommendations’ specifically focusing on NH residents and awareness of vaccination of residents as an important infection control measure. There are, however, still NHs with no residents vaccinated; thus a substantial effort is still required in order to reach the overall target of 75%.

Vaccination of HCWs has been shown to play an important role in controlling transmission of influenza to residents [5]. A review from the Norwegian Knowledge Center for the Health Services came to the conclusion that influenza vaccination in HCWs reduced the risk of acquiring influenza- like illness in residents by 50% [11]. On the other hand, a recent Cochrane review shows that, when using laboratory confirmed influenza as an endpoint [12], there is insufficient evidence to prove the effect of vaccinating HCWs in relation to the transmission of influenza to residents. The Cochrane review, however, did not take influenza like illness (ILI) and all-cause mortality of residents into consideration, The impact of vaccinating HCWs has been discussed in several articles. Potter [13] and Carman [14] took ILI into consideration in their studies and found a significant association between HCWs’ vaccination against influenza and reduced rates of mortality in residents of NHs, showing how important it is to vaccinate HCWs. HCW absence from work owing to influenza is another important issue and has been studied by several authors [13,15-17]. These studies demonstrate reductions in work-time lost owing to illness among vaccinated HCWs. Moreover, in all of these studies, unvaccinated HCWs with influenza continued to work while symptomatic and may have infected residents. WHO also recommends the vaccination of HCWs against seasonal influenza in two other respects: One, as an effective measure in decreasing the extent of seasonal influenza and two, as a measure towards maintaining an active workforce during influenza epidemics [5,6]. As studies in other European countries have shown [18-22], we expected a low VC. Our results are markedly lower than the results of the VENICE project on influenza VC among all HCWs (12%), which is based on a telephone survey of the general Norwegian population and the numbers of influenza vaccines sold [8]. Most HCWs, however, are employed by hospitals, which conduct more comprehensive infection control. It is thus not unlikely that VC among HCWs in hospitals is higher than in NHs. We therefore suppose that our findings are more likely to reflect the actual VC among HCWs in NHs.

In order to raise VC among HCWs, several hospitals in the USA have already made seasonal influenza vaccination mandatory for HCWs with direct patient contact [23,24]. In the Netherlands too, mandatory vaccination programs are being discussed owing to low VC among HCWs working in NHs. The authors of a Dutch study concerning the ethical aspects of mandatory vaccination conclude that NHs have a moral responsibility to implement vaccination programs with a view to getting their HCWs to accept voluntary vaccination, but that this does not exclude the possibility of introducing mandatory vaccination if VC rates don’t rise [25].

The results of our study show that having to pay for the vaccine does not affect the VC of HCWs while having annual vaccination campaigns does lead to increases in VC. In the Netherlands, vaccination campaigns have also been found to increase VC while, interestingly, having to pay for the vaccine as a HCW in a hospital actually decreased VC [22].

The low VC among HCWs warrants studies to see why they are so reluctant to be vaccinated. Specifically, research into the reason for low VC in HCWs, as well as into behavioral changes that are positively associated with VC are needed. In the Netherlands, studies have revealed a number of important, significant attitudes held by HCWs in relation to influenza vaccination. These include the responsibility not to harm patients, the knowledge that the vaccine has high efficacy, and the belief that they (HCWs) are in a high risk group in relation to contracting influenza infection [22,25,26].

One limitation in our study is the low response rate. We initially intended to reach all NHs through the generic e-mail addresses of the municipalities, as we do not have access to direct telephone and e-mail lists. More than half of the NHs did not reply and any generalization from the results should therefore be treated with caution. While the e-mail modality is fast and convenient and allows for the respondents to collect the requested data, it may also result in lower response rates compared to telephone modality interviews as undertaken in Venice [8,27]. We did not, however, find an association between the response rate per county and the VC. All the same, non-participating NHs could be vaccinating fewer residents, resulting in an overestimation of the VC and introducing bias towards “the best in the class”.

The extent to which NHs document vaccination is unknown and we are unable to state if, and how much, this influences our results.

## Conclusion

From the results of our study, we are able to conclude that the median influenza VC among residents of the participating NHs is not much lower than recommended. On the other hand, influenza VC among HCWs in the participating NHs is exceedingly low. VC in HCWs was significantly higher when NHs had an annual vaccination campaign. We recommend immediate measures be implemented to increase VC in residents and, especially, in HCWs. These measures should encompass vaccination campaigns and studies to identify potential barriers to vaccination. In order to achieve more comprehensive and accurate data in future, a direct communication channel with NHs should be established.

## Competing interests

The authors declare that they have no competing interests.

## Authors’ contributions

HB was responsible for the idea, planning, conceptualizing and implementation of the study, interpreting the data and writing the manuscript. JVB made substantial contributions to the planning and conceptualizing of the study, interpretation of results, and drafting and reviewing of the manuscript. SHH was involved in planning the study and contributed to the drafting and revision of the manuscript in relation to important subject specific content. MRB contributed to the drafting and revision of the manuscript in relation to important subject specific content. All authors read and approved the final manuscript.

## Pre-publication history

The pre-publication history for this paper can be accessed here:

http://www.biomedcentral.com/1471-2458/14/434/prepub

## Supplementary Material

Additional file 1 **Copy of the questionnaire sent to the municipalities for distribution to all their nursing homes (NHs).** Translated from the Norwegian original. Click here for file
