# Supplementary material for: Vaccination coverage for seasonal influenza among residents and health care workers in Norwegian nursing homes during the 2012/13 season, a cross-sectional study
Source: BMC Public Health. 2014 May 9;14:434. doi: 10.1186/1471-2458-14-434 (PMC4049507; doi:10.1186/1471-2458-14-434)
Supplement: Additional file 1 — Copy of the questionnaire sent to the municipalities for distribution to all their nursing homes (NHs). Translated from the Norwegian original. [file 1471-2458-14-434-S1.pdf]

Copy of the questionnaire sent to the municipalities for distribution to all their nursing homes (NHs)

Translated from the original Norwegian.

The Norwegian Institute of Public Health invites all nursing homes (NHs) in Norway to participate in a study on vaccination coverage for seasonal influenza this year.

The questionnaire should be completed by the NH manager and/or the responsible GP, at the NH.

Before you start with the questionnaire we kindly ask you to have the information on number of long-term care residents, healthcare workers and data on their vaccination status to hand. This will make it easier for you to complete the questionnaire. It will take you between 5-10 minutes to complete the questionnaire.

We thank all the NH in advance for your participation and effort.

Neither the names nor the municipality of the NHs will be published. Names mentioned in the questionnaire will only be used for quality control to avoid f. example double registration. Results will only be published on county and country level.

The questionnaire should be answered

1. What is the name of your NH?
   Name:
2. In which municipality is your NH situated?
   Municipality:
3. In which county is your NH situated?
   County:
4. How many long term care residents are there in your institution?
   Number of residents:
5. How many Healthcare workers (HCWs) with direct patient contact work in your facility?
   Number of HCWs:
6. Do you have seasonal Influenza vaccination campaigns?
   Yes/No/Don't know
7. How many of your long-term residents have been vaccinated against seasonal influenza for this season (winter 2012/2013)?
   Number of residents vaccinated:
8. Does your institution offer seasonal vaccination to your employees every year?
   Yes/No/Don't know
9. Does your institution offer seasonal vaccination to your employees free of charge?
   Yes/No/Don't know

- If vaccination is not free of charge, how much does the employee have to contribute? Amount of contribution (Nkr):

10. How many HCWs have been vaccinated against seasonal influenza for this season (winter 2012/2013)?
    Number of HCWs vaccinated:
